# Human Cdc14B Promotes Progression through Mitosis by Dephosphorylating Cdc25 and Regulating Cdk1/Cyclin B Activity

**DOI:** 10.1371/journal.pone.0014711

**Published:** 2011-02-17

**Authors:** Indra Tumurbaatar, Onur Cizmecioglu, Ingrid Hoffmann, Ingrid Grummt, Renate Voit

**Affiliations:** 1 Molecular Biology of the Cell II, German Cancer Research Centre, DKFZ-ZMBH Alliance, Heidelberg, Germany; 2 Mammalian Cell Cycle Control Mechanisms, German Cancer Research Centre, Heidelberg, Germany; CNRS UMR6543, Université de Nice, Sophia Antipolis, France

## Abstract

Entry into and progression through mitosis depends on phosphorylation and dephosphorylation of key substrates. In yeast, the nucleolar phosphatase Cdc14 is pivotal for exit from mitosis counteracting Cdk1-dependent phosphorylations. Whether hCdc14B, the human homolog of yeast Cdc14, plays a similar function in mitosis is not yet known. Here we show that hCdc14B serves a critical role in regulating progression through mitosis, which is distinct from hCdc14A. Unscheduled overexpression of hCdc14B delays activation of two master regulators of mitosis, Cdc25 and Cdk1, and slows down entry into mitosis. Depletion of hCdc14B by RNAi prevents timely inactivation of Cdk1/cyclin B and dephosphorylation of Cdc25, leading to severe mitotic defects, such as delay of metaphase/anaphase transition, lagging chromosomes, multipolar spindles and binucleation. The results demonstrate that hCdc14B-dependent modulation of Cdc25 phosphatase and Cdk1/cyclin B activity is tightly linked to correct chromosome segregation and bipolar spindle formation, processes that are required for proper progression through mitosis and maintenance of genomic stability.

## Introduction

Cdk1/cyclin B is the central kinase that promotes entry into and progression through early stages of mitosis by triggering a variety of mitotic events, such as breakdown of the nuclear envelope, condensation of chromosomes, and assembly of the mitotic spindle [1]–[3]. During interphase, Cdk1 activity is downregulated by Wee1- and Myt1-dependent phosphorylation of conserved threonine T14 and tyrosine Y15 residues in the ATP-binding domain of Cdk1 [1]. Activation of Cdk1/cyclin B is achieved by a complex mitotic entry network, which consists of several feedback loops. Through a central feedback loop Cdk1 is activated by Cdc25-dependent dephosphorylation at pT14/pY15. Once activated, Cdk1/cyclin B phosphorylates Cdc25 phosphatases as well as Myt1 and Wee1 kinases, augmenting Cdc25 activity and repressing Myt1 and Wee1. Cdk1/cyclin B activity and mitotic entry are further controlled by additional superimposed feedback mechanisms upregulating the mitotic kinases Plk1 and Aurora A, and the Aurora/Plk1 activator Bora, coupling mitotic entry to centrosome maturation, and stimulating expression of proteins of the mitotic entry network, such as cyclin B [4].

Cdc25 phosphatases are highly conserved among eukaryotes. The three isoforms of mammalian Cdc25, A, B and C, are all regulated by reversible phosphorylation, phosphorylation affecting their enzymatic activity, intracellular localisation and stability [5]–[9]. Consistent with the notion that phosphorylation-dependent activation of Cdc25s is part of the central positive feedback amplification loop that increases Cdk1/cyclin B activity and promotes entry into mitosis, ablation of Cdc25A and B delays G_2_/M transition [8]–[10], whereas overexpression induces premature activation of Cdk1 and accelerates entry into mitosis [8], [10]–[11]. In contrast to Cdc25A and B, Cdc25C alone is not sufficient for mitotic entry [10]. During M/G_1_ transition down-regulation of Cdk1 activates Wee1 and Myt1 kinases and inhibits Cdc25 phosphatases [12]. Thus, completion of mitosis depends on dephosphorylation and inactivation of Cdc25 and Cdk1/cyclin B as well as reversal of Cdk1/cyclin B-dependent phosphorylations.

In budding yeast, yCdc14 antagonizes the action of mitotic Cdks, triggering the degradation of mitotic cyclins and regulating a variety of mitotic events, such as spindle dynamics, rDNA segregation and cytokinesis [13]–[18]. yCdc14 is sequestered in the nucleolus during interphase and is activated upon release from the nucleolus at anaphase [19]–[20]. Mammalian cells express two isoforms of Cdc14, the cytoplasmic phosphatase hCdc14A and the nucleolar phosphatase hCdc14B [21], both of which target proteins that are phosphorylated by proline-directed kinases [22]. Despite their evolutionary conservation, the physiological role of mammalian Cdc14 phosphatases is poorly understood. Several functions have been assigned to human Cdc14A (hCdc14A), including centrosome splitting, mitotic spindle formation, and chromosome segregation [23]–[24]. Few physiological substrates of hCdc14A have been identified, e.g. SIRT2 [25], Erk3 [26], and the Rab5 GTPase-activator RN-tre [27]. Mammalian Cdc14B (hCdc14B), like its yeast counterpart, is sequestered in nucleoli during interphase and released during mitosis [23]–[24], [28]. Release from the nucleolus is also triggered by the G_2_-DNA damage checkpoint leading to hCdc14B-induced activation of APC/C^Cdh1^ [29]. Moreover, both hCdc14A and hCdc14B have been implicated in centriole amplification [30] and DNA repair [31]. Given the evolutionary conservation of basic biological mechanisms, one would anticipate that hCdc14B, like yCdc14, controls processes that trigger progression through mitosis. In support of this view, hCdc14B has been shown to modulate the assembly and disassembly of the mitotic spindle by bundling and stabilizing microtubules, yet apparently independent of its catalytic activity [28]. In addition, hCdc14B reverses mitotic phosphorylations on SIRT2 and Skp2, thereby triggering proteasome-dependent degradation of these proteins, and promoting mitotic exit and entry into G_1_-phase [25], [32].

In this study, we have examined the role of hCdc14B during mitosis. We show that hCdc14B is associated with nucleolar chromatin during interphase, released at prometaphase and rebound in early G_1_. RNAi-induced depletion of hCdc14B causes errors in chromosome segregation, metaphase delay, multipolar spindles, and cell death due to accumulation of multiple mitotic defects. hCdc14B dephosphorylates and inactivates the mitotic inducer Cdc25, enabling efficient inactivation of Cdk1 at late M-phase. Together, our results show for the first time that hCdc14B serves an important role in mitotic progression, regulating the activity of Cdc25s and Cdk1/cyclin B.

## Results

### Unscheduled Expression of hCdc14B disturbs Timing of Mitosis

**Figure 1:**
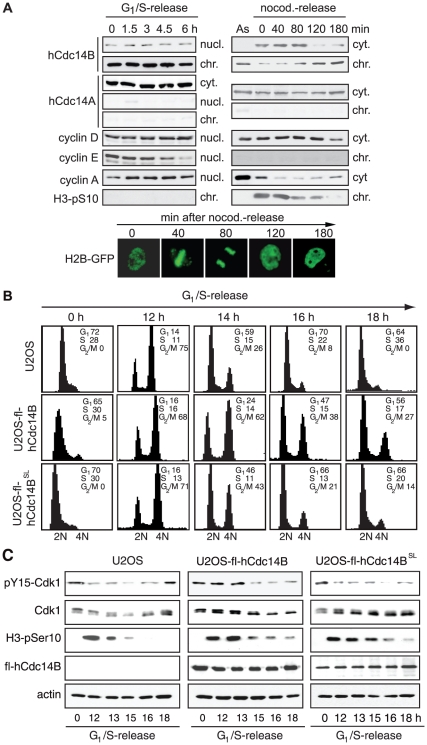
Overexpression of hCdc14B alters progression through mitosis. **A**. hCdc14B is released from chromatin in early mitosis and is rebound in late M/early G_1_. HeLa cells expressing histone H2B-GFP (HeLa Kyoto) were arrested at G_1_/S by double thymidine-block (left) or at prometaphase of mitosis by nocodazole (right), and released for the indicated times. Release from nocodazole (nocod.) arrest was monitored visualizing H2B-GFP labelled chromatin (bottom panel). Synchronized cells were fractionated into cytoplasm (cyt.), nucleoplasm (nucl.) and chromatin (chr.), and analyzed on immunoblots for the proteins indicated (top panels). **B**. Overexpression of hCdc14B prolongs G_2_/M-phase. FACS of U2OS, U2OS-fl-hCdc14B and U2OS-fl-hCdc14B^SL^ cells following release from G_1_/S (0 h) upon induction of hCdc14B expression by doxycyclin (Fig. S1). Cell numbers were calculated using the Cell Quest software program. The percentage of cells at the indicated cell cycle phases is presented. **C**. Overexpression of hCdc14B affects mitotic progression. Immunoblots showing the abundance of specific cell cycle marker proteins at distinct time points (h) after release from G_1_/S.

Immunofluorescence studies have demonstrated that hCdc14B localizes within nucleoli in interphase cells [23], [28]. Fractionation of extracts from synchronized HeLa Kyoto cells that stably express GFP-tagged histone H2B (H2B-GFP) showed that hCdc14B was associated with chromatin during interphase and was released into the soluble fraction at prometaphase (Fig. 1A). Re-association with chromatin started around telophase and was completed after decondensation of chromatin in early G_1_ (Fig. 1A, right panels and bottom images). In contrast, virtually all hCdc14A was contained in the soluble cytoplasmic fraction throughout the cell cycle and was not detectable in the nucleoplasmic or chromatin fraction (Fig. 1A). The cell cycle-dependent, dynamic interaction of hCdc14B with nucleolar chromatin suggests that nucleolar sequestration serves a regulatory function in cycling cells similar to yeast Cdc14, restricting hCdc14B activity from early mitosis to telophase.

To examine a potential role of hCdc14B in mitosis, we generated U2OS cell lines that conditionally express Flag-tagged hCdc14B (U2OS-fl-hCdc14B) or the catalytically inactive mutant (U2OS-fl-hCdc14B^SL^). Clonal cell lines were synchronized by double thymidine block, hCdc14B expression was induced concomitant with release from the G_1_/S arrest (Supp. Fig. S1) and cell cycle progression was determined by flow cytometry analyses (FACS) (Fig. 1B). The FACS profiles were similar in parental and fl-hCdc14B cell lines until the cells reached the G_2_/M-phase (12 h after release), indicating that overexpression of wildtype or mutant hCdc14B had not affected progression through S- and G_2_-phase. However, re-entry into the next G_1_-phase was delayed in cells expressing fl-hCdc14B. While in parental cells progression through G_2_/M-phase and entry into the next G_1_-phase was completed after 16–18 h, a significant fraction of cells expressing fl-hCdc14B (38%) and fl-hCdc14B^SL^ (21%) were still in G_2_/M 16 h after release indicating aberrant timing of mitosis.

To investigate whether delayed re-entry into G_1_ was due to delay in mitotic entry or prolonged mitosis, we monitored the phosphorylation state of pY15-Cdk1 on immunoblots. In parental and U2OS-fl-hCdc14B^SL^ cells, pY15-Cdk1 phosphorylation was low at 12 h after release from G_1_/S, consistent with a timely entry into mitosis. In contrast, in cells overexpressing wildtype fl-hCdc14B, pY15 levels remained elevated up to 13 h (Fig. 1C). This suggests that increased levels of hCdc14B prevented timely dephosphorylation and activation of Cdk1, thereby delaying entry into mitosis. Consistent with the FACS data showing prolonged G_2_/M and delayed entry into G_1_, there was no *de novo* phosphorylation of Cdk1 up to 18 h after release from G_1_/S in fl-hCdc14B cells. Moreover, cell proliferation was reduced in clonal cell lines expressing fl-hCdc14B, proliferation inversely correlating with the level of ectopic fl-hCdc14B (Supp. Fig. S2A and B). In contrast, overexpression of mutant fl-hCdc14B^SL^ did not impair dephosphorylation of Cdk1 on Y15, but mitotic phosphorylation of H3-pSer10 was extended over a longer period of time (3 h compared to parental cells) (Fig. 1C), indicating that either progression through mitosis or mitotic exit were delayed.

### Depletion of hCdc14B Impairs Progression through Mitosis

**Figure 2:**
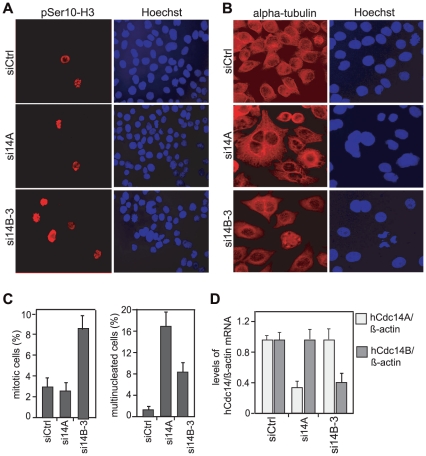
Silencing of hCdc14B leads to enrichment of mitotic cells. HeLa Kyoto cells were reverse transfected twice with a control-siRNA pool (siCtrl) or siRNAs that target hCdc14A (si14A) [24, Reference S1] or hCdc14B (si14B-3). During the second transfection equal numbers of cells were seeded onto coverslips, and 24 h after the second transfection cells were subjected to immunofluorescence microscopy and analysis of hCdc14 expression. **A**, **B**. Representative immunofluorescence images of HeLa Kyoto cells transfected with siCtrl, si14A, or si14B-3. Mitotic cells were visualized by immunostaining with histone H3-phospho-Serine-10 (pSer10-H3) antibodies (red) (A), multinucleated cells by staining with anti-alpha-tubulin antibodies (red) (B). DNA was counterstained with Hoechst 33342. **C**. Frequencies of mitotic and multinucleated cells. The data shown here represent means (±SD) of three independent experiments derived from counting 400 cells each. **D**. hCdc14A and hCdc14B expression is reduced by the corresponding siRNAs. The graphs represent the mean levels (±SD) of hCdc14A-mRNA (light bar) and hCdc14B-mRNA (dark bar) normalized to β-actin-mRNA as determined by RT-qPCR in three independent experiments.

Given that overexpression of hCdc14B impaired progression through mitosis, we next analyzed the mitotic phenotype caused by ablation of hCdc14B. Treatment of HeLa Kyoto cells with hCdc14A- or hCdc14B-specific siRNAs for 72 h reduced the level of hCdc14A- or hCdc14B-mRNAs to 35–60% (Fig. 2D). Immunofluorescence analysis of cycling cells treated with Cdc14A- or Cdc14B si-RNAs revealed that pSer10-H3-positive, mitotic cells were enriched up to 3-fold upon depletion of hCdc14B compared to cells treated with control siRNA or hCdc14A-specific siRNA (Fig. 2A and C). hCdc14A- and hCdc14B-specific siRNAs caused a drop in the overall cell number (data not shown), suggesting that both isoforms are required for proper cell proliferation. Consistent with results of Mailand et al. [24], the number of bi- and multinucleated cells was significantly increased in hCdc14A-deficient cells (Fig. 2B and C). Notably, binucleation was also observed upon knockdown of hCdc14B, albeit at lower frequency compared to hCdc14A-depleted cells (Fig. 2B and C). This suggests that knockdown of hCdc14A primarily perturbed cytokinesis without affecting progression through mitosis, whereas depletion of hCdc14B also impaired events required for progression through mitosis depletion increasing the amount of mitotic cells.

**Figure 3:**
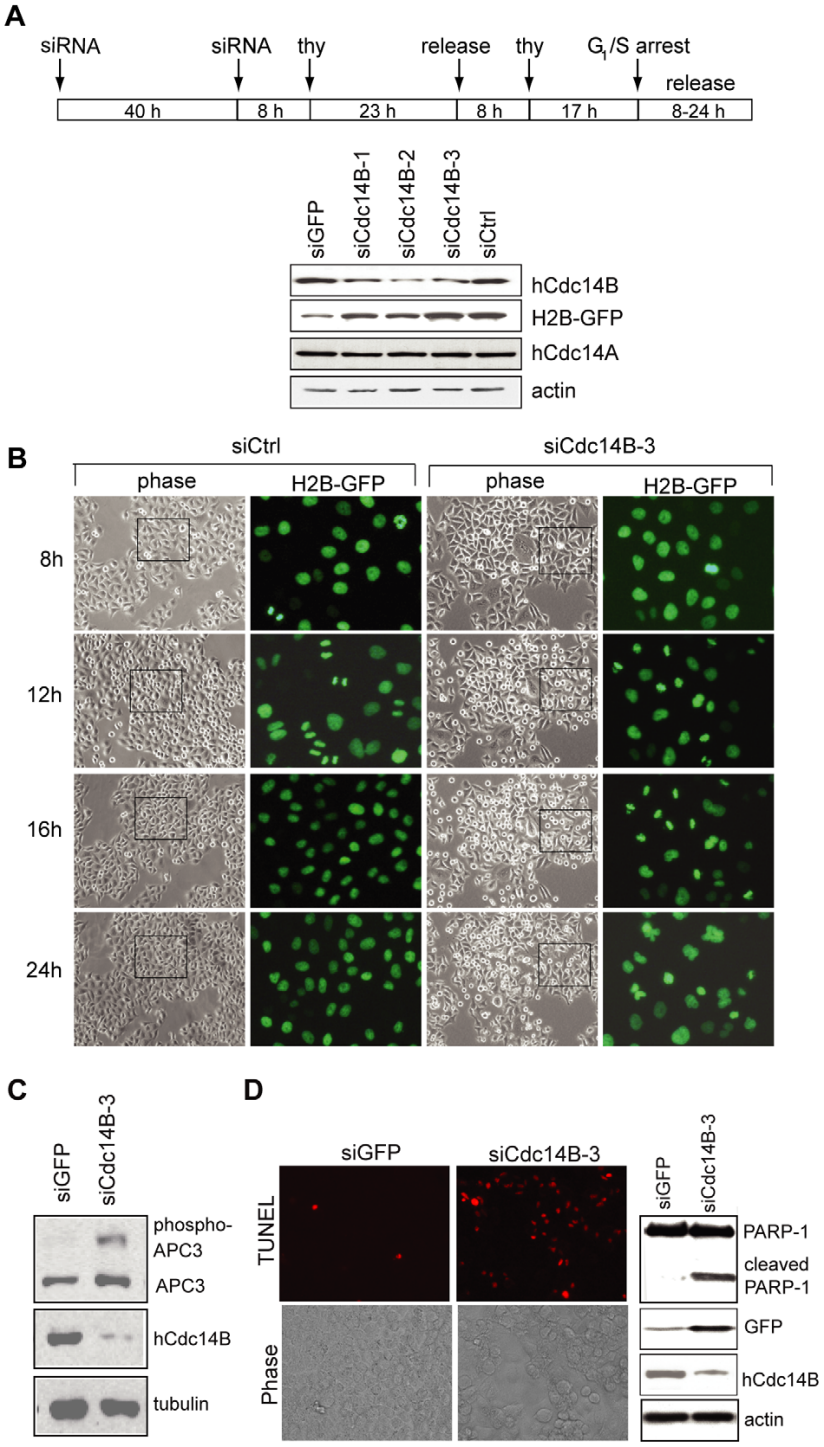
Depletion of hCdc14B impairs progression through mitosis and triggers apoptosis. **A**. Cell synchronization and specificity of hCdc14B knockdown by siRNAs. HeLa Kyoto cells were reverse transfected with a control siRNA pool (siCtrl), siRNAs that target GFP-mRNA (siGFP), or three different regions of hCdc14B-mRNA (siCdc14B-1, -2, -3; see Table S1 for sequence information) and synchronized as shown in the scheme. 8 h after release from the second G_1_/S arrest, cells were lysed and the amount of hCdc14B, hCdc14A, H2B-GFP, and actin was analyzed on immunoblots. **B**. Phase contrast and H2B-GFP fluorescence images of HeLa Kyoto cells that were transfected with control-siRNA (siCtrl) or hCdc14B-siRNA (siCdc14B-3) and released from G_1_/S according to the scheme above. Before the second G_1_/S arrest, equal numbers of cells were seeded onto coverslips. The GFP images represent magnifications of regions marked by rectangles in the phase contrast images. **C**. Western blot analysis of Cdc27/APC3, hCdc14B, and β-tubulin in lysates from cells treated with GFP-specific (siGFP) or hCdc14B-specific (siCdc14B-3) siRNAs. The hyperphosphorylated form of Cdc27/APC3 is indicated. **D**. hCdc14B depletion induces apoptosis. siRNA transfections and cell synchronization was done as described above. Before the second thymidine block, equal numbers of cells were seeded on coverslips and apoptosis was monitored 24 h after release from the second G_1_/S-arrest in control (siGFP) or hCdc14B-depleted (siCdc14B-3) HeLa Kyoto cells by TUNEL staining (left) and on immunoblots monitoring caspase-induced proteolysis of PARP-1 (right).

We next examined mitotic progression upon depletion of hCdc14B in synchronized HeLa Kyoto cells (Fig. 3A). After release from the second G_1_/S-arrest, progression into mitosis was monitored by live microscopy visualizing chromosome condensation via H2B-GFP-labeled chromatin. Representative images of the phenotypic responses from RNAi with an hCdc14B-specific siRNA are shown in Figure 3B. Cells entered mitosis 12 h after release, regardless whether or not hCdc14B was depleted. While most of control cells re-entered a new division cycle at 16 h after release from G_1_/S, a significant proportion (48%) of hCdc14B-depleted cells was enriched in metaphase (Fig. 3B and Table 1), suggesting that hCdc14B activity is required for successful completion of mitosis. Consistent with the delay of metaphase to anaphase transition, prolonged mitotic hyperphosphorylation of Cdc27/APC3 [33] was observed (Fig. 3C). Moreover, 24 h after release a large number of hCdc14B-siRNA treated cells stained positive in the TUNEL assay with cleavage of PARP-1 (Fig. 3D and Table 1), indicating that the strong delay in mid-/late-mitosis has triggered apoptosis. Again and consistent with knockdown of hCdc14B in non-synchronized cells (Fig. 2), the amount of bi- and multinucleated cells was increased (Supp. Fig. S3 and Table 1). Together, these results strongly suggest that downregulation of hCdc14B impairs metaphase-anaphase transition mitotic delay leading to cell death.

**Table 1:** Knockdown of hCdc14B leads to increase of the mitotic index, multinucleation and apoptosis.

Release from G_1_/S (h)	siCtrl: mitotic index (%)	siCdc14B-3: mitotic index (%)
8	9.4±2.7	8.6±2.5
10	11.3±3.8	11.0±4.7
12	56.6±6.8	51.4±12.8
14	28.3±4.2	50.5±10.6
16	16.9±4.0	47.9±8.8

Release from G_1_/S (h)	siCtrl: multinucleated cells (%)	siCdc14B-3: multinucleated cells (%)
8	0.5±0.15	6.5±3.0

Release from G_1_/S (h)	siCtrl: TUNEL positive cells (%)	siCdc14B-3: TUNEL positive cells (%)
24	5.4±1.2	42.6±8.5

HeLa Kyoto cells transfected with hCdc14B-specific siRNA-3 (siCdc14B-3) or non-targeting siRNAs (siCtrl) were synchronized as shown in Fig. 3A and B. Mitotic cells with condensed chromatin were counted 8–16 h after release from G_1_/S, multinucleated cells were counted 8 h after release, and apoptotic cells were visualized by TUNEL staining 24 h after release. Numbers represent the mean levels (±SD) from three independent experiments comprising counts of 400 cells each.

### Silencing of hCdc14B Causes Chromosome Mis-segregation and Multipolar Spindles

**Figure 4:**
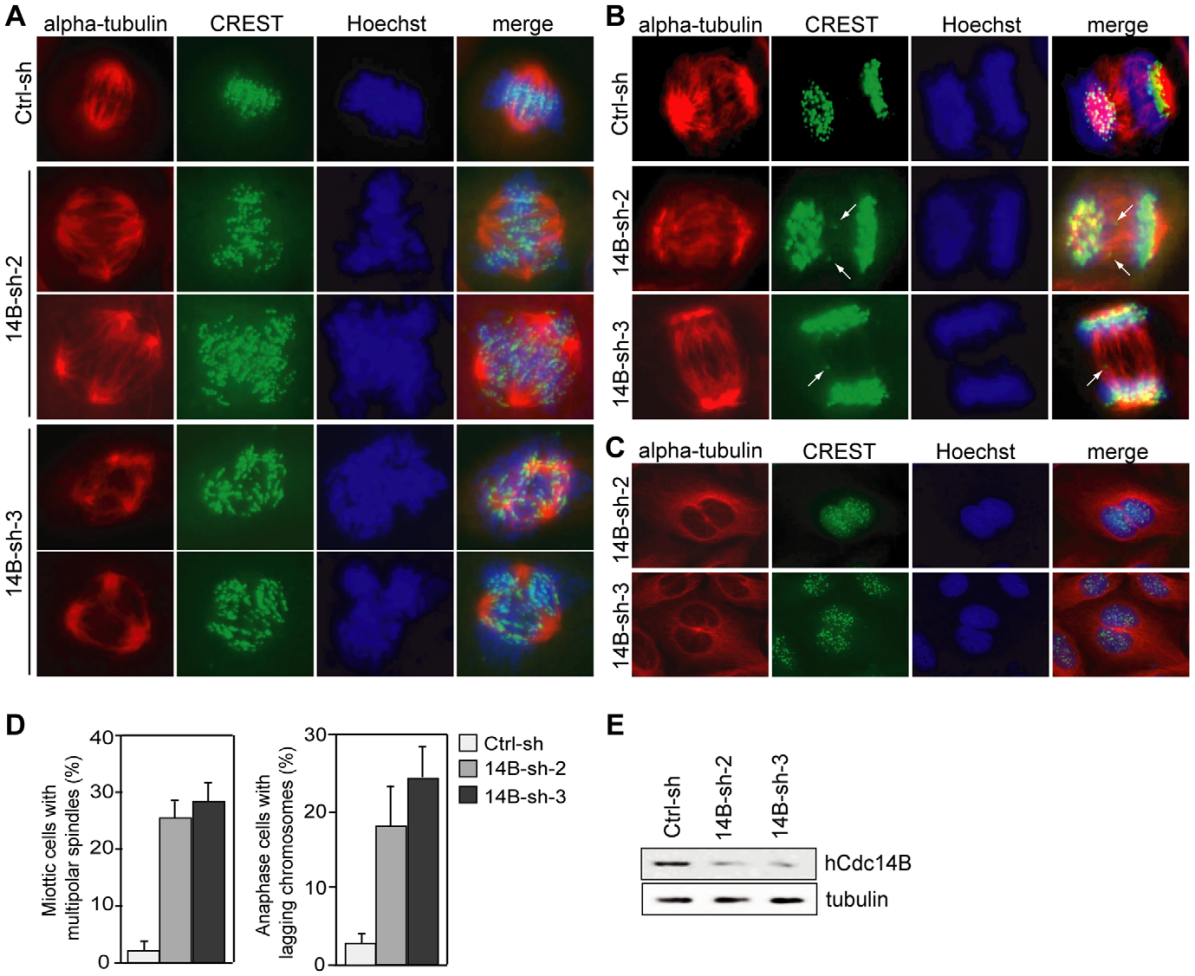
Downregulation of hCdc14B leads to multipolar mitotic spindles, lagging chromosomes, and binucleation. U2OS cells were transfected with pTER plasmids expressing two different hCdc14B-specific shRNAs (14B-sh-2, 14B-sh-3) or a control shRNA (Ctrl-sh). 72 h after transfection, cells were processed for immunofluorescence using anti-alpha-tubulin and Cy3-coupled anti-mouse antibodies (red), and CREST antiserum (kinetochores) and FITC-coupled anti-human antibodies (green). DNA was stained with Hoechst 33342. Representative examples of multipolar mitotic cells (**A**), bipolar mitotic cells with lagging anaphase chromosomes (arrow) (**B**), and binucleated cells (**C**) are shown. **D**. Frequencies of multipolar spindles and lagging anaphase chromosomes as visualized by staining with anti-alpha-tubulin and CREST. The numbers are derived from three independent experiments, each count comprising 60 mitotic cells. **E**. Western blot of hCdc14B after transfection with pTER-ctrl-shRNA (Ctrl-sh), pTER-Cdc14B-2 (14B-sh-2), and pTER-Cdc14B-3 (14B-sh-3) for 72 h. Decrease of hCdc14B expression was normalized to β-tubulin.

To investigate whether silencing of hCdc14B had perturbed spindle assembly or chromosome segregation, immunofluorescence microscopy was performed with CREST and anti-alpha-tubulin antibodies. Immunostaining of mitotic spindles in U2OS cells depleted of hCdc14B by hCdc14B-shRNA-2 or hCdc14B-shRNA-3 (Fig. 4E) revealed multi-polar spindles at frequencies that were significantly higher (24–28%) than in cells transfected with an unrelated Ctrl-shRNA (2.5%). Most of the aberrant spindles were tri- or tetrapolar (Fig. 4A and D), although some cells with even more than 5 spindle poles were observed. In addition, hCdc14B-depletion increased the number of bipolar anaphase cells with lagging chromosomes (18–25%), indicative of chromosome missegregation (Fig. 4B and D). Again, while frequency of binucleation was low in control cells, it was significantly elevated in hCdc14B-depleted cells (Fig. 4C). These analyses indicate that silencing of hCdc14B caused problems in proper segregation of chromosomes, which in turn may explain the delay in mid-mitosis observed in HeLa Kyoto cells depleted of hCdc14B (see Fig. 3), and caused defects in bipolar mitotic spindle assembly, which is consistent with previous studies demonstrating a role of hCdc14B in bundling of microtubules [28] and in centriole amplification [30].

### hCdc14B Regulates Cdk1/Cyclin B Activity

**Figure 5:**
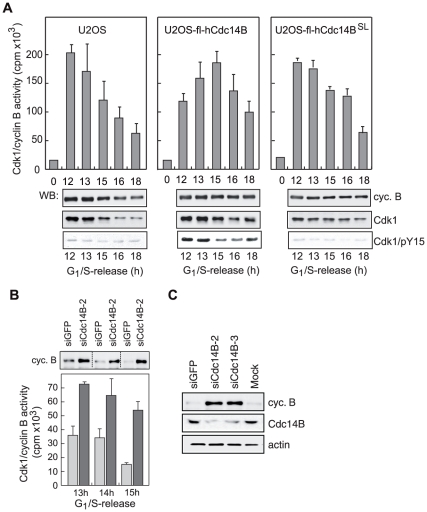
hCdc14B regulates Cdk1/cyclin B activity. **A**. Overexpression of hCdc14B alters Cdk1/cyclin B activity. Cdk1/cyclin B was immunopurified from U2OS, U2OS-fl-hCdc14B and U2OS-fl-hCdc14B^SL^ cells that were synchronized at G_1_/S (0 h) and released for the indicated times. Kinase activity was assayed *in vitro* using Cdk1-specific peptides and ^32^P-ATP. Values were normalized to the activity of Cdk1/cyclin B in G_1_/S cells. The Western blots show the amount of immunoprecipitated cyclin B, Cdk1 and Cdk1/pY15. **B**. Depletion of hCdc14B increases Cdk1/cyclin B activity. Cells were transfected with GFP- (siGFP, light bars) or hCdc14B-specific siRNA-2 (siCdc14B-2, dark bars) and arrested by double thymidine block. Kinase activity of immunopurified Cdk1/cyclin B was assayed *in vitro* at the indicated times (h) after release from G_1_/S. The immunoblot on the top shows the amounts of cyclin B used in the assays. **C**. Cyclin B is stabilized upon knockdown of hCdc14B. Cells were transfected with siRNAs against GFP (siGFP), hCdc14B (siCdc14B-2 and siCdc14B-3) or left untransfected (mock), synchronized by double thymidine block, released from G_1_/S for 16 h and analyzed on immunoblots.

Reversal of mitotic Cdk1-dependent phosphorylations and inhibition of Cdk1 are hallmarks of Cdc14 function in yeast [18], [34]. To decipher a functional link between human Cdc14B and Cdk1/cyclin B, we examined whether changes in hCdc14B levels would affect Cdk1 activity. For this, we compared activation of Cdk1/cyclin B in U2OS cell lines conditionally expressing fl-hCdc14B or fl-hCdc14B^SL^. After induction of wildtype or mutant fl-hCdc14B in G_1_/S arrested cells (0 h), kinase activity of immunopurified Cdk1/cyclin B was assayed at distinct times after release. In parental cells, Cdk1/cyclin B activity reached maximal levels after 12–13 h, and then declined because of cyclin B degradation (Fig. 5A, left). In contrast and consistent with data shown in Figure 1B, activation of Cdk1 was delayed in cells expressing fl-hCdc14B, reaching maximal levels at 15 h, which declined more slowly than in parental cells (Fig. 5A, middle). The delay in Cdk1 activation correlated with elevated levels of pY15 (Fig. 5A, middle). Cdk1 activity was high over an extended period in cells expressing the catalytically inactive mutant fl-hCdc14B^SL^ (Fig. 5A, right), suggesting that hCdc14B phosphatase is required for timely inactivation of Cdk1/cyclin B.

To further corroborate this result, Cdk1/cyclin B activity was measured in synchronized cells depleted of hCdc14B. Treatment with hCdc14B-siRNAs increased Cdk1/cyclin B activity almost 2-fold compared to control (Fig. 5B). Consistent with hCdc14B promoting activation of APC^Cdh1^ [29], cyclin B levels were elevated upon depletion of hCdc14B correlating with increased Cdk1 activity (Fig. 5B and C) implying that degradation of cyclin B was impaired upon hCdc14B depletion. Significantly, hCdc14B did not affect Cdk1/cyclin B activity *in vitro* (Supp. Fig. S4), suggesting that hCdc14B does not directly target Cdk1/cyclin B, but rather activates proteins and regulators that act upstream of Cdk1/cyclin B.

### hCdc14B Dephosphorylates and Inactivates Cdc25

**Figure 6:**
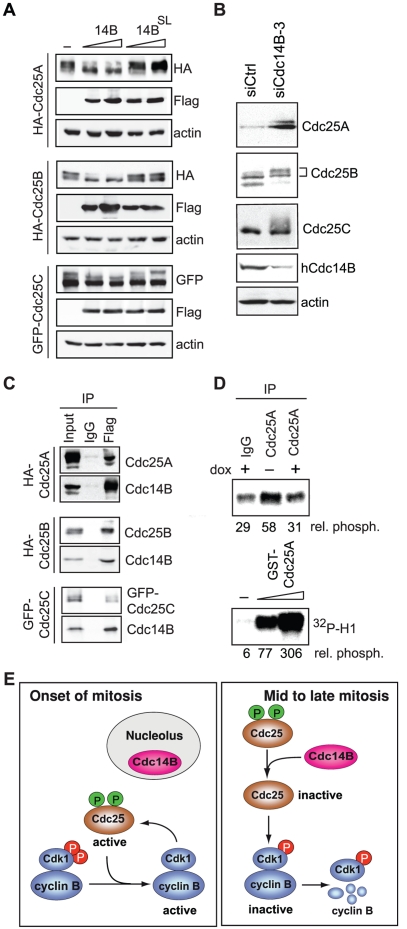
hCdc14B dephosphorylates Cdc25. **A**. hCdc14B dephosphorylates Cdc25 *in vivo*. Immunoblots of nocodazole-arrested HEK293T cells co-expressing fl-hCdc14B or fl-hCdc14B^SL^ and HA-Cdc25A, HA-Cdc25B or GFP-Cdc25C. The upper bands correspond to hyperphosphorylated Cdc25 proteins. **B**. Cdc25 phosphatases are hyperphosphorylated in cells depleted of hCdc14B. HeLa Kyoto cells transfected with control (siCtrl) or hCdc14B-specific siRNA-3 (siCdc14B-3) were released from G_1_/S for 18 h, and analyzed on Western blots. **C**. hCdc14B interacts with Cdc25 isoforms *in vivo*. Immunoblots showing co-precipitation of Cdc25 isoforms with fl-hCdc14B^SL^ from 293T cells co-expressing HA-Cdc25A, HA-Cdc25B, GFP-Cdc25C, and fl-hCdc14B^SL^. **D**. Overexpression of hCdc14B leads to inactivation of Cdc25A. Cdc25A was immunoprecipitated from doxycyclin-treated (dox) or untreated U2OS-fl-hCdc14B cells, incubated with Cdk1/cyclin B from S-phase cells, and kinase activity of Cdk1/cyclin B was monitored by *in vitro* phosphorylation of histone H1 (top). As a control, activation of Cdk1/cyclin B was monitored using recombinant GST-Cdc25A (bottom). Quantification of histone H1 phosphorylation was performed with a PhosphorImager and is shown below. **E**. Model depicting the role of hCdc14B during mitosis. Entry into mitosis requires a positive feedback loop, involving Cdk1-dependent activation of Cdc25 phosphatases and Cdc25-dependent dephosphorylation of Cdk1 on pT14/Y15. Before onset of mitosis, hCdc14B is sequestered within the nucleolus, preventing premature dephosphorylation and inactivation of Cdc25 proteins. From prometaphase until late mitosis, hCdc14B is released from nucleolar chromatin, leading to inactivation of Cdc25s, which triggers inhibitory phosphorylation of Cdk1 and inactivation of Cdk1/cyclin B.

Phosphorylation of Cdc25 promotes activation of Cdk1, inactivation of Cdc25 correlating with repression of Cdk1 activity [35]. We therefore investigated whether hCdc14B targets Cdc25 phosphatases, dephosphorylation of Cdc25 proteins by hCdc14B promoting inactivation of Cdk1 and completion of mitosis. Indeed, wildtype but not mutant hCdc14B or alkaline phosphatase (CIAP) efficiently dephosphorylated all three isoforms of Cdc25 *in vitro* (Supp. Fig. S5A and B). To prove that hCdc14B also targeted Cdc25s *in vivo*, cells co-expressing fl-hCdc14B and the different isoforms of Cdc25 were arrested in mitosis, and phosphorylation of Cdc25A, B and C was analyzed on immunoblots. Consistent with the *in vitro* data, fl-hCdc14B dephosphorylated all three isoforms of Cdc25, shifting the hyperphosphorylated to the faster migrating hypophosphorylated form (Fig. 6A). Notably, siRNA-mediated depletion of hCdc14B increased the level of hyperphosphorylated, active Cdc25A, B and C (Fig. 6B) demonstrating a link between hCdc14B and the phosphorylation level of all three Cdc25 isoforms.

hCdc14A has been shown to reverse Cdk1/cyclin B-dependent phosphorylation of Cdc25A at S115 and S320 [36]. Cdc25A is phosphorylated at multiple sites, which is required for full Cdc25A activity during early mitosis [7]–[8]. To analyze whether the two human Cdc14 isoforms A and B target the same residues on Cdc25A, GST-Cdc25A was phosphorylated with Cdk1/cyclin B immunopurified from mitotic extracts followed by incubation with either recombinant hCdc14A or hCdc14B (Supp. Fig. S6A). Semi-quantitative mass spectrometry analysis detected several tryptic phospho-peptides of Cdc25A that contained S/T-P directed phosphosites, e.g. serine 18, 40, 88, 116, 261, and 283 (Supp. Fig. S6B). Comparison of removal of these phosphorylations by hCdc14A or hCdc14B revealed that one phosphosites, e.g. S40, was equally dephosphorylated by both isoforms, whereas other sites were preferentially dephosphorylated by either hCdc14A (phospho-S116) or hCdc14B (phospho-S88 and phospho-S261) (Supp. Figure S6B). This result confirms previous data from Esteban et al [36] showing that hCdc14A dephosphorylates phospho-serine-116 (equivalent to phospho-serine-115) of hCdc25A. Moreover, the analysis suggests that hCdc14A- and B target sites are not fully overlapping.

The functional interplay between Cdc25 and hCdc14B suggests that the two phosphatases may interact in vivo with each other. Indeed, co-immunoprecipitation experiments demonstrated that hCdc14B interacts with all three Cdc25 isoforms (Fig. 6C). Notably, these interactions are direct as shown by GST-pull down assays using GST-Cdc25 proteins and in vitro translated hCdc14B (Supp. Fig. S7). To demonstrate that hCdc14B inactivates Cdc25, we immunopurified Cdc25A from non-induced and induced U2OS-fl-hCdc14B cells, and assayed Cdc25A activity by measuring its ability to activate Cdk1 that was isolated from S-phase cells. As shown in Figure 6D, overexpression of fl-hCdc14B reduced Cdk1-dependent phosphorylation of histone H1 to background levels, indicating that hCdc14B has inactivated Cdc25A (upper panel, lane 3). In contrast, Cdc25A isolated from non-induced cells was twice as active towards stimulating Cdk1 activity as shown by increased phosphorylation of histone H1 (upper panel, lane 2). Collectively, the results indicate that hCdc14B inactivates Cdc25 phosphatases, thereby counteracting unscheduled activation of Cdk1, which in turn promotes completion of mitosis.

## Discussion

Though several studies have demonstrated the essential role of yeast Cdc14 for reversal of Cdk-mediated mitotic phosphorylations and mitotic exit [16], it remained to be established whether mammalian Cdc14 homologues serve a similar function. In this study, we present several lines of evidence showing that human Cdc14B plays a key role in regulation of Cdk1/cyclin B activity and progression through mitosis. First, activation of Cdk1/cyclin B and entry into mitosis is delayed in cells overexpressing hCdc14B, the delay of mitotic entry correlating with inhibitory phosphorylation of Y15/Cdk1. Second, hCdc14B dephosphorylated and inactivated mitotic Cdc25 proteins, the phosphatases that remove inhibitory phosphates from T14/Y15 and activate Cdk1 at G_2_/M transition. Third, depletion of hCdc14B by siRNA led to hyperphosphorylation of Cdc25, increased Cdk1 activity, mitotic defects such as delayed anaphase chromosomes and multipolar spindles, and finally to mitotic arrest and cell death.

Similar to yeast, hCdc14B is associated with nucleolar chromatin only during interphase, and is released into the cytoplasm from prometaphase on [19], [23], [28]. Thus, nucleolar sequestration represents an evolutionarily conserved mechanism that controls hCdc14B function enabling hCdc14B to target substrates at defined stages during mitosis and shielding target proteins from being dephosphorylated during interphase. In support of this view, elevated levels of hCdc14B perturbed nucleolar sequestration and delayed entry into mitosis. Notably, it was recently shown that perturbation of cell cycle progression by genotoxic stress also induces nucleolar release of hCdc14B correlating with hCdc14B-dependent activation of APC/C^Cdh1^ and G_2_-arrest [29].

G_2_/M transition is tightly controlled by a complex mitotic entry network activation of Cdk1 representing the pivotal event. To achieve full activity, Cdc25 phosphatases are upregulated at G_2_/M by Cdk1- and Plk-dependent phosphorylations, initiating an auto-amplification loop that ensures rapid mitotic entry. Vice versa, completion of mitosis relies on inactivation of Cdk1/cyclin B as well as reversal of mitotic phosphorylations. In mammalian cells more than one thousand phosphosites are generated by proline-directed kinases during mitosis, mostly by Cdk1 [37]. The mitotic phosphatases that dephosphorylate Cdk1 substrates are poorly characterized. In yeast, Cdc14 is a component of two consecutive mitotic exit systems, FEAR and MEN that activate Cdc14 leading to inhibition of mitotic Cdk1 activity and reversal of Cdk1-dependent phosphorylations [13], [38]. In this study we have shown that hCdc14B plays a pivotal role in dephosphorylating and inactivating Cdc25 proteins leading to decrease of Cdk1/cyclin B activity. Inhibition of Cdk1/cyclin B is largely accomplished by proteasomal degradation of cyclin B through APC^Cdc20^ and APC^Cdh1^ [2]. Besides this well-established pathway Cdk1/cyclin B is also inactivated in a non-proteolytic fashion at onset of anaphase by transient inhibitory Wee1-mediated phosphorylation of Y15 on Cdk1 [39]. Since inhibitory phosphorylation on Cdk1 is counteracted by Cdc25 proteins, phosphatases that target and repress Cdc25 proteins in mid-mitosis have been postulated [40]. Our data demonstrate that hCdc14B is a candidate phosphatase, since (i) hCdc14B dephosphorylates all three isoforms of Cdc25, and (ii) knockdown of hCdc14B delays inactivation of mitotic Cdk1 correlating with high level of hyperphosphorylated, active Cdc25s. Therefore, hCdc14B may act in two ways to inactivate Cdk1/cyclin B at late mitosis, e.g. by dephosphorylating and activating the APC co-activator Cdh1 [29] and by enabling Cdk1/Y15 phosphorylation by Wee1 upon dephosphorylation and inactivation of Cdc25 phosphatases.

Our results are consistent with the model depicted in Figure 6E. Entry into mitosis depends on the establishment of a positive feedback loop involving Cdk1-dependent activation of Cdc25 phosphatases and Cdc25-dependent dephosphorylation of Cdk1 on pT14/pY15. At this stage of the cell cycle, nucleolar sequestration of hCdc14B prevents premature dephosphorylation and inactivation of Cdc25s. Transient release from nucleolar chromatin at prometaphase enables hCdc14B to interact with Cdc25 proteins. Dephosphorylation by hCdc14B inactivates Cdc25 phosphatases, allowing *de novo* phosphorylation of Cdk1 on Y15 and inactivation of Cdk1. Consistently depletion of hCdc14B prevents timely inactivation of Cdk1/cyclin B, leading to delay of metaphase and severe mitotic defects. As in human cells a large number of proteins are phosphorylated by Cdk1/cyclin B during mitosis [37], prolonged Cdk1 activity and failure of reversal of Cdk1 phosphorylations will affect a wide range of mitotic processes. Therefore, depletion of hCdc14B caused severe mitotic defects, including chromosome missegregation, erroneous mitotic spindle formation and binucleation.

So far, it was not known whether the hCdc14A and B isoforms contribute to site-specific dephosphorylation of Cdc25 proteins. According to our semi-quantitative mass spectrometric analysis substrate specificity differs between hCdc14A and hCdc14B at least to some extent. Some phosphorylations of Cdc25A, e.g. pS88 and pS261, were removed by hCdc14B, but were retained after incubation with hCdc14A. This suggests that the hCdc14 isoforms target different sites within substrates and are functionally not fully redundant. Functional analysis of mitotic regulation by phosphatases has been hampered by the complex phenotypes associated with inactivation of mitotic phosphatases, making it difficult to distinguish between primary and secondary effects [38]. Nevertheless, the phenotypes resulting from knockdown of hCdc14B or hCdc14A are distinct and consistent with hCdc14A playing primarily a role in centrosome splitting, and hCdc14B promoting primarily progression through mitosis and chromosome segregation.

Studies on the role of hCdc14B in cell cycle regulation have been controversial, depending on the experimental systems used, e.g. depletion by RNAi, overexpression or gene knockout in cultured cell lines. hCdc14B has been implicated in centriole amplification [30], mitotic exit [25], M/G_1_ transition [32], G_2_-DNA damage check point activation [29], DNA repair [31], bundling and stabilizing microtubules, and assembly of the mitotic spindle [28]. In contrast to our results, a recent study by Berdougo et al [40] showed no obvious mitotic defects in human colorectal carcinoma HCT116 cells in which the hCdc14B gene has been genetically inactivated. This obvious discrepancy may be due to mechanisms that established during selection of the somatic knockout cells compensating loss of hCdc14B. Requirement for hCdc14B may also vary between different cell lines. We observed that hCdc14B levels are high in HeLa cells but low in HCT116 cells (unpublished data). A recent RNAi screen identified PP2A-B55a as a phosphatase that promotes late mitosis and mitotic exit suggesting that other phosphatases may compensate loss of hCdc14B in a cell-type specific manner [41]. In addition, hominoids contain another hCdc14B-like gene, known as hCdc14C [42]. Therefore it cannot be ruled out, that other phosphatases functionally replace hCdc14B in different cell lines to trigger inactivation of Cdk1 in mid mitosis. Notably, expression of hCdc14C is inhibited by the siRNAs used in our study but not by the hCdc14B knockout strategy used by Berdougo et al [40]. Therefore, the differences in mitotic phenotypes may be attributed to hCdc14C replacing the function of hCdc14B. This would also explain the observation that Cdc14B-specific antibodies detected the hCdc14 protein in wildtype as well as in Cdc14B^−/−^ cells [40]. Clearly, further systemic approaches are required to identify additional physiological substrates of hCdc14B and to elucidate the processes that link hCdc14B activity to cell division.

## Materials and Methods

### Plasmids and Antibodies

Human cDNAs encoding hCdc14A and hCdc14B (accession numbers AF064102.1 and AF064104.1) were cloned into pTOPO-CR2.1 (Clontech), pRc/CMV-Flag, pET-28a, pGEX-2T, and pcDNA4/TO (Invitrogen). Point mutations (C326S and A328L in hCdc14B^SL^) within the catalytic domain of hCdc14B were introduced by PCR. All PCR-generated fragments were verified by DNA sequencing. Plasmids encoding Cdc25A, B and C have been described [9], [43]–[44]. The shRNA expression vector pTER^+^ [45, Reference S2] was used to insert duplex oligonucleotides corresponding to different regions of the hCdc14B coding region (see Table S2).

Antibodies used were anti-Cdc14B (Zymed), anti-Flag (M2, Sigma), anti-HA (12CA5), anti-GFP (sc-8334 FL), anti-actin (C4), anti-H3-phospho-Ser10 (Upstate), anti-Cdk1 (sc-054), anti-cyclin B (sc-GNS1), anti-cyclin E (sc-C19), anti-cyclin A (Oncogene, AB-2), anti-cyclin D (sc-HD11), anti-Cdk1 phospho-specific (Calbiochem), anti-Cdc25A (sc F-6), anti-Cdc25B (sc C-20), anti-Cdc25C (sc C-20), anti-APC3/Cdc27 (BD Biosciences), anti-tubulin (Sigma B-5-1-2), and CREST autoimmune antibodies. Polyclonal antibodies against hCdc14A produced in rabbits were purified using GST-hCdc14A coupled to Affigel 10 (Bio-Rad).

### Cell Culture and Transfections

HeLa cells expressing H2B-GFP (Kyoto) were cultured in DMEM/10% FCS containing 500 µg/ml of G418. U2OS and 293T cells were obtained from ATCC. To generate U2OS-fl-hCdc14B cell lines, U2OS cells were co-transfected with pcDNA/6TR (Clontech) and pcDNA/TO-fl-hCdc14B or pcDNA/TO-fl-hCdc14B^SL^. Clones were selected in tetracycline-free medium supplemented with zeocin (250 µg/ml) and blasticidin (5 µg/ml). For fl-hCdc14B expression, cells were treated with doxycyclin (1 µg/ml). Cells were synchronized by a double thymidine-block, and fl-hCdc14B expression was induced by addition of doxycyclin 8 h before cells were released from the second thymidine-block. Cells transfected with shRNA expression plasmids were selected in the presence of zeocin (250 µg/ml) [45]. siRNA duplexes (40–100 nM) were reverse transfected with lipofectamin 2000 (Invitrogen). siRNA and shRNA sequences are given in Table S1 and S2. Cdc14 transcripts were measured by reverse transcription using random hexamer primers and quantitative real-time PCR (LightCycler 480, Roche) essentially as described [46]. Data were normalized to the level of actin-mRNA. Primer sequences are given in Table S3.

### Fractionation of Cell Extracts

Cells were fractionated as described [47] with minor modifications. Buffer B contained 10 mM HEPES, pH 7.9, 150 mM NaCl, 3 mM EDTA, 0.2 mM EGTA, and 1 mM DTT. Chromatin-bound proteins were extracted with SDS-sample buffer supplemented with 2 mM MgCl_2_ 0.5 mM PMSF and protease inhibitors (Complete, Roche). After sonication (Bioruptor), samples were treated with benzonase (10 U/100 µl, 15 min at RT) and clarified by centrifugation.

### Immunocytochemistry

Cells grown on poly-lysine coated coverslips were fixed with 2% paraformaldehyde, permeabilized with ice-cold methanol, and incubated with the indicated first antibodies and fluorescence-labelled secondary antibodies (Invitrogen, Dianova). DNA was stained using Hoechst 33342. Apoptotic cells were detected by TUNEL assay using the In Situ Cell Death Detection Kit (Roche).

### Purification of Recombinant Phosphatases

GST and recombinant GST-tagged proteins were expressed in E. coli Codon Plus-RP cells (Stratagene), affinity-purified over glutathione-Sepharose and eluted according to standard procedures.

### Protein Interaction Assays

Purified GST-tagged isoforms of Cdc25 were incubated with *in vitro* translated ^35^S-labeled hCdc14B for 4 h at 4°C in buffer AM-120 (120 mM KCl, 20 mM Tris-HCl pH 7.9, 5 mM MgCl_2_, 0.2 mM EDTA, 10% glycerol, 0.5 mM EDTA), supplemented with 0.2% NP-40 and protease inhibitors (Complete, Roche). For co-immunoprecipitation of hCdc14B and Cdc25s, 1.5×10^6^ U2OS cells were transfected with the corresponding expression plasmids, treated with 10 µM MG132 for 6 h, and lysed in buffer AM-120 containing 0.5% NP-40, 10% glycerol, DNase I (200 U/ml), RNase A (100 µg/ml), and protease inhibitors (Complete, Roche). After incubation with 10 µl (1∶1 slurry) of anti-Flag (M2)-agarose (Sigma), immunoprecipitated proteins were analyzed on immunoblots.

### Enzymatic Assays

Cdk1/cyclin B and Cdc25 activity were analyzed as described [48], [49]. Cdc14B activity was assayed using pNPP as substrate. Briefly, hCdc14B was incubated for 60 min at 30°C in phosphatase assay buffer (50 mM Tris-HCl, pH 6.8, 2 mM EDTA, 0.01% Triton-X-100, 2% glycerol, 150 mM NaCl, 5 mM DTT) containing 20 mM p-nitrophenylphosphate (pNPP). Substrate turnover was measured in a spectrophotometer at 405 nm. To assay dephosphorylation of Cdc25, bead-bound GST-Cdc25 proteins were phosphorylated with immunopurified Cdk1/cyclin B complexes [48] or by incubation with extracts from mitotic HeLa cells (30 µg protein, 30 min, 30°C) in 50 mM Tris-HCl, pH 7.5, 10 mM MgCl_2_, 1 mM EGTA, 20 mM β-glycerophosphate, 1 mM DTT, 10 µM ATP and 5 µCi ^32^P-ATP. After washing, ^32^P-labeled GST-Cdc25 isoforms were incubated for 45 min at 37°C with equal enzymatic units of recombinant hCdc14B, hCdc14B^SL^ or CIAP (Roche) in phosphatase assay buffer.

Cdc25A activity was measured essentially as described [49]. Cdk1/cyclin B was immunopurified from S-phase cells using anti-cyclin B antibodies. U2OS-fl-hCdc14B cells were cultured in the absence or presence of doxycyclin (2 µg/ml, 8 h). Cdc25A was immunoprecipitated from equal amounts of cells and incubated with bead-bound Cdk1/cyclin B in 100 µl of phosphatase assay buffer (15 min, 30°C). Control reactions contained the same amount of cell lysate and rabbit IgGs (Dianova). After washing, beads were incubated for 15 min at 30°C in 50 µl histone H1 kinase assay buffer (50 mM Tris-HCl pH 7.5, 10 mM MgCl_2_, 1 mM DTT, 5 µg histone H1 (Roche), 50 µM ATP, 5 µCi ^32^P- ATP), subjected to SDS-PAGE, and radioactive proteins were detected by PhosphorImaging.

### Analysis of Phosphopeptides by Mass Spectrometry

GST-Cdc25A was phosphorylated with immunopurified Cdk1/cyclin B for 1 h at 30°C in kinase buffer supplemented with 50 µM ATP in the absence or presence of radioactive ^32^P-ATP. GST-Cdc25A beads were washed three times in high salt buffer (20 mM Tris-HCl, pH 7.9, 1 M KCl, 5 mM MgCl_2_, 0.2 mM EDTA, 10% glycerol, 0.5 mM DTT), equilibrated twice in phosphatase assay buffer, and incubated for 1 h at 30°C in phosphatase assay buffer containing equal phosphatase activities (1.2 U) of hCdc14A or hCdc14B, or no phosphatase for control. GST-Cdc25A beads were again washed in high salt buffer, equilibrated in 50 mM ammonium bicarbonate, and proteins were digested with trypsin overnight at 37°C. The resulting tryptic peptides were analyzed by nanoLC ESI-MS/MS. ESI-MS/MS analysis and database search were performed essentially as described [50].

## Supporting Information

References S1 (0.04 MB DOC)

Figure S1 Doxycyclin (dox)-induced expression of fl-hCdc14B and fl-hCdc14BSL in clonal U2OS-fl-hCdc14B cells. Cells were synchronized at G1/S by a double thymidine-block and released in the presence of dox (1 μg/ml) for the indicated time (h). Fl-hCdc14B expression was analyzed by Western blotting using anti-Cdc14B antibodies.
(0.96 MB EPS)

Figure S2 Prolonged overexpression of fl-hCdc14B in U2OS cells increases the cell doubling time. A. Fl-hCdc14B expression was induced in three different clonal U2OS-fl-hCdc14B cell lines by doxycyclin (1 μg/ml), and fl-hCdc14B expression was monitored over 48 h by Western blotting. B. Overexpression of fl-hCdc14B in U2OS-fl-hCdc14B cell lines prolongs the cell doubling time. Equal amounts of U2OS-fl-hCdc14B cells of clones 6, 7, and 13 were seeded in 6 cm culture dishes and incubated in the absence or presence of doxycyclin (dox, 1 μg/ml). After 56 h cells were harvested and counted. The bars show the relative mean amount of cells (±SD) obtained from the non-induced culture (gray bars) and the induced culture (black bars). The values are derived from 3 independent experiments.
(1.88 MB EPS)

Figure S3 Depletion of hCdc14B leads to bi- and multinucleation of cells. Cells transfected with GFP-specific siRNA (siGFP) or hCdc14B-siRNA-3 (siCdc14B-3) were analyzed for multinucleated cells. Representative phase contrast images are shown. Arrows indicate multinucleated cells.
(2.79 MB EPS)

Figure S4 hCdc14B downregulates Cdk1/cyclin B activity in vivo but not in vitro. A. HEK293T cells overexpressing fl-hCdc14B or fl-hCdc14BSL were treated with nocodazole for 22 h, and the kinase activity of immunopurified Cdk1/cyclin B was measured in vitro in the presence of Cdk1-specific peptides as described in Figure 5A. B. hCdc14B does not affect the activity of Cdk1/cyclin B in vitro. Cdk1/cyclin B activity was determined after incubation with GST (1 μg) or GST-hCdc14B (1 and 2 μg) for 30 min at 30°C (left panel). Right panel: In vitro phosphatase assay showing the activity of GST-hCdc14B in the presence of 20 mM pNPP as substrate. Values are from three independent experiments (mean ±SD).
(0.40 MB EPS)

Figure S5 hCdc14B dephosphorylates Cdc25 proteins in vitro. A. GST-tagged Cdc25A, B, and C were immobilized on Glutathione-Sepharose and phosphorylated in vitro using extracts from mitotic HeLa cells and 32P-ATP. Phosphorylated Cdc25 was incubated with increasing amounts of GST-hCdc14B or the corresponding activity of calf intestine alkaline phosphatase (CIAP, 0.025, 0.05, and 0.125 U), or was left untreated (-). After SDS-PAGE, GST-Cdc25 and GST-hCdc14B proteins were stained with Coomassie Blue (C panels), and the level of Cdc25 phosphorylation was monitored by PhosphorImaging (32P panels). B. GST-tagged Cdc25A and B were immobilized on Glutathione-Sepharose and phosphorylated in vitro using Cdk1/cyclin B complexes purified from Sf9 cells and 32P-ATP. After washing, the beads were incubated for 45 min at 30°C with 1 μg of GST-hCdc14B, 1 μg of the phosphatase-dead mutant GST-hCdc14BSL, or left untreated. Reactions were stopped, samples separated by 8% SDS-PAGE, and stained with Coomassie Blue (bottom panels). 32P-labelled Cdc25 (top panels) was detected by autoradiography.
(2.03 MB EPS)

Figure S6 Mass spectrometry-based identification of GST-Cdc25A peptides phosphorylated by Cdk1/cyclin B and dephosphorylated by hCdc14A/B. Recombinant GST-Cdc25A was phosphorylated in vitro by Cdk1/cyclin B immunopurified from mitotic HeLa cell extracts in the absence or presence of 32P-ATP, followed by incubation with recombinant hCdc14A or B. A. 32P-phosphorylated GST-Cdc25A was incubated with 1.2 or 0.6 U of recombinant His-hCdc14A or B or was left untreated (-). Following SDS-PAGE, GST-Cdc25A phosphorylation was monitored by PhosphorImaging (left panel). Phosphatase activity of recombinant His-hCdc14B (white bars) and His-hCdc14A (grey bars) was determined in vitro using pNPP as substrate (right panel). Values are derived from three independent assays (mean ±SD). B. Summary of tryptic GST-Cdc25A phospho-peptides identified with nanoLC ESI-MS/MS and Mascot search engine (top panel). Schematic presentation of the relative abundance of GST-Cdc25A phosphopeptides in the absence of hCdc14A or B (black bar) and after incubation with hCdc14B (white bar) or hCdc14A (grey bar).
(0.53 MB EPS)

Figure S7 hCdc14B interacts with all three isoforms of Cdc25. GST or GST-Cdc25A, B, and C were incubated for 4 h at 4°C with 35S-labeled fl-hCdc14B. Bead-bound proteins were separated by SDS-PAGE and visualized by PhosphorImaging. The first lane shows 10% of the input proteins.
(0.37 MB EPS)

Table S1 Sequences of the siRNAs used in this study. All sequences are written from 5 to 3 orientation. Chemically synthesized siRNA duplexes were obtained from Eurogentec (siCdc14B), MWG (siGFP), and Dharmacon (siCdc14A and siCtrl corresponding to the non-targeting siRNA pool 1). Sequences of the non-targeting siRNA pool 1 are available from Dharmacon.
(0.03 MB DOC)

Table S2 Sequences of shRNAs used in this study. DNA oligonucleotides (given in 5 to 3 orientation) encoding sense and antisense strands were cloned into the shRNA expression vector pTER as described [S2]. shRNA targeting sequences are highlighted in bold.
(0.03 MB DOC)

Table S3 Sequences of primers used for RT-qPCR in this study.
(0.03 MB DOC)

## References

[1] Malumbres M, Barbacid M (2005). Mammalian cyclin-dependent kinases.. Trends Biochem Sci.

[2] Morgan DO (1995). Principles of CDK regulation.. Nature.

[3] Nigg EA (2001). Mitotic kinases as regulators of cell division and its checkpoints.. Nat Rev Mol Cell Biol.

[4] Lindqvist A, Rodríguez-Bravo V, Medema RH (2009). The decision to enter mitosis: feedback and redundancy in the mitotic entry network.. J Cell Biol.

[5] Boutros R, Dozier C, Ducommun B (2006). The when and wheres of CDC25 phosphatases.. Curr Opin Cell Biol.

[6] Nilsson I, Hoffmann I (2000). Cell cycle regulation by the Cdc25 phosphatase family.. Prog Cell Cycle Res.

[7] Trinkle-Mulcahy L, Lamond AI (2006). Mitotic phosphatases: no longer silent partners.. Curr Opin Cell Biol.

[8] Mailand N, Podtelejnikov AV, Groth A, Mann M, Bartek J (2002). Regulation of G2/M events by Cdc25A through phosphorylation-dependent modulation of its stability.. EMBO J.

[9] Lammer C, Wagerer S, Saffrich R, Mertens D, Ansorge W (1998). The cdc25B phosphatase is essential for the G_2_/M phase transition in human cells.. J Cell Sci.

[10] Lindqvist A, Källstrom H, Lundgren A, Barsoum E, Rosenthal CK (2005). Cdc25B cooperates with Cdc25A to induce mitosis but has a unique role in activating cyclin B1-Cdk1 at the centrosome.. J Cell Biol.

[11] Timofeev O, Cizmecioglu O, Settele F, Kempf T, Hoffmann I (2010). Cdc25 phosphatases are required for timely assembly of CDK1-cyclin B at the G2/M transition.. J Biol Chem.

[12] Potapova TA, Daum JR, Byrd KS, Gorbsky GJ (2009). Fine tuning the cell cycle: Activation of the cdk1 inhibitory phosphorylation pathway during mitotic exit.. Mol Biol Cell.

[13] D'Amours D, Amon A (2004). At the interface between signaling and executing anaphase - Cdc14 and the FEAR network.. Genes Dev.

[14] Higuchi T, Uhlmann F (2005). Stabilization of microtubule dynamics at anaphase onset promotes chromosome segregation.. Nature.

[15] Jaspersen SL, Charles JF, Morgan DO (1999). Inhibitory phosphorylation of the APC regulator Hct1 is controlled by the kinase Cdc28 and the phosphatase Cdc14.. Curr Biol.

[16] Stegmeier F, Amon A (2004). Closing mitosis: the functions of the Cdc14 phosphatase and its regulation.. Ann Rev Genet.

[17] Sullivan M, Higuchi T, Katis VL, Uhlmann F (2004). Cdc14 phosphatase induces rDNA condensation and resolves cohesin-independent cohesion during budding yeast anaphase.. Cell.

[18] Visintin R, Craig K, Hwang ES, Prinz S, Tyers M (1998). The phosphatase Cdc14 triggers mitotic exit by reversal of Cdk-dependent phosphorylation.. Mol Cell.

[19] Shou W, Seol JH, Shevchenko A, Baskerville C, Moazed D (1999). Exit from mitosis is triggered by Tem1-dependent release of the phosphatase Cdc14 from nucleolar RENT complex.. Cell.

[20] Visintin R, Hwang ES, Amon A (1999). Cfi1 prevents premature exit from mitosis by anchoring Cdc14 phosphatase in the nucleolus.. Nature.

[21] Li L, Ljungman M, Dixon JE (2000). The human Cdc14 phosphatases interact with and dephosphorylate the tumor suppressor protein p53.. J Biol Chem.

[22] Gray CH, Good VM, Tonks NK, Barford D (2003). The structure of the cell cycle protein Cdc14 reveals a proline-directed protein phosphatase.. EMBO J.

[23] Kaiser BK, Zimmermann ZA, Charbonneau H, Jackson PK (2002). Disruption of centrosome structure, chromosome segregation, and cytokinesis by misexpression of human Cdc14A phosphatase.. Mol Biol Cell.

[24] Mailand N, Lukas C, Kaiser BK, Jackson PK, Bartek J (2002). Deregulated human Cdc14A phosphatase disrupts centrosome separation and chromosome segregation.. Nat Cell Biol.

[25] Dryden SC, Nahhas FA, Nowak JE, Goustin AS, Tainsky MA (2003). Role for human SIRT2 NAD-dependent deacetylase activity in control of mitotic exit in the cell cycle.. Mol Cell Biol.

[26] Tanguay PL, Rodier G, Meloche S (2010). C-terminal domain phosphorylation of ERK3 controlled by Cdk1 and Cdc14 regulates its stability in mitosis.. Biochem J.

[27] Lanzetti L, Margaria V, Melander F, Virgili L, Lee MH (2007). Regulation of the Rab5 GTPase-activating protein RN-tre by the dual specificity phosphatase Cdc14A in human cells.. J Biol Chem.

[28] Cho HP, Liu Y, Gomez M, Dunlap J, Myers M (2005). The dual-specificity phosphatase CDC14B bundles and stabilizes microtubules.. Mol Cell Biol.

[29] Bassermann F, Frescas D, Guardavaccaro D, Busino L, Peschiaroli A (2008). The Cdc14B-Cdh1-Plk1 axis controls the G2 DNA-damage-response checkpoint.. Cell.

[30] Wu J, Cho HP, Johnson DK, Dunlap J, Liu Y (2008). Cdc14B depletion leads to centriole amplification, and its overexpression prevents unscheduled centriole duplication.. J Cell Biol.

[31] Mocciaro A, Berdougo E, Zeng K, Black E, Vagnarelli P (2010). Vertebrate cells genetically deficient for Cdc14A or Cdc14B retain DNA damage checkpoint proficiency but are impaired in DNA repair.. J Cell Biol.

[32] Rodier G, Coulombe P, Tanguay PL, Boutonnet C, Meloche S (2008). Phosphorylation of Skp2 regulated by CDK2 and Cdc14B protects it from degradation by APC(Cdh1) in G1 phase.. EMBO J.

[33] Kraft C, Herzog F, Gieffers C, Mechtler K, Hagting A (2003). Mitotic regulation of the human anaphase-promoting complex by phosphorylation.. EMBO J.

[34] Wolfe BA, Gould KL (2004). Fission yeast Clp1p phosphatase affects G2/M transition and mitotic exit through Cdc25p inactivation.. EMBO J.

[35] Donzelli M, Draetta GF (2003). Regulating mammalian checkpoints through Cdc25 inactivation.. EMBO Rep.

[36] Esteban V, Vázquez-Novelle MD, Calvo E, Bueno A, Sacristán MP (2006). Human Cdc14A reverses CDK1 phosphorylation of Cdc25A on serines 115 and 320.. Cell Cycle.

[37] Olsen JH, Vermeulen M, Santamaria A, Kumar C, Miller ML (2010). Quantitative phosphoproteomics reveals widespread full phosphorylation site occupancy during mitosis.. Sci Signal.

[38] Bollen M, Gerlich DW, Lesage B (2009). Mitotic phosphatases: from entry guards to exit guides.. Trends Cell Biol.

[39] D'Angiolella V, Palazzo L, Santarpia C, Costanzo V, Grieco D (2007). Role for non-proteolytic control of M-phase promoting factor activity at M-phase exit.. PLoS ONE.

[40] Berdougo E, Nachury MV, Jackson PK, Jallepalli PV (2008). The nucleolar phosphatase Cdc14B is dispensable for chromosome segregation and mitotic exit in human cells.. Cell Cycle.

[41] Schmitz MHA, Held M, Janssens V, Hutchins JRA, Hudecz O (2010). Live-cell imaging RNAi screen identifies PP2A-B55a and importin-b1 as key mitotic exit regulators in human cells.. Nat Cell Biol.

[42] Rosso L, Marques AC, Weier M, Lambert N, Lambot MA (2008). Birth and rapid subcellular adaptation of a hominoid-specific CDC14 protein.. PLoS Biol.

[43] Hassepass I, Voit R, Hoffmann I (2003). Phosphorylation at serine 75 is required for UV-mediated degradation of human Cdc25A phosphatase at the S-phase checkpoint.. J Biol Chem.

[44] Karlsson C, Katich S, Hagting A, Hoffmann I, Pines J (1999). Cdc25B and C differ markedly in their properties as initiators of mitosis.. J Cell Biol.

[45] van de Wetering M, Oving I, Muncan V, Fong MJP, Brantjes H (2003). Specific inhibition of gene expression using a stably integrated, inducible small-interfering-RNA vector.. EMBO Rep.

[46] Hoppe S, Bierhoff H, Cado I, Weber A, Tiebe M (2009). AMP-activated protein kinase adapts rRNA synthesis to cellular energy supply.. Proc Natl Acad Sci USA.

[47] Watrin E, Schleiffer A, Tanaka K, Eisenhaber F, Nasmyth K (2006). Human Scc4 is required for cohesin binding to chromatin, sister-chromatid cohesion, and mitotic progression.. Curr Biol.

[48] Heix J, Vente A, Voit R, Budde A, Michaelidis TM (1998). Mitotic silencing of human rRNA synthesis: inactivation of the promoter selectivity factor SL1 by cdc2/cyclin B-mediated phosphorylation.. EMBO J.

[49] Hassepass I, Hoffmann I (2004). Assaying Cdc25 phosphatase activity.. Methods Mol Biol.

[50] Schokraie W, Hotz-Wagenblatt A, Warnken U, Mali B, Frohme M (2010). Proteomic analysis of tardigrades: toward a better understanding of molecular mechanisms by anhydrobiotic organisms.. PLoS ONE.

