# Supplementary material for: Human Cdc14B Promotes Progression through Mitosis by Dephosphorylating Cdc25 and Regulating Cdk1/Cyclin B Activity
Source: PLoS One. 2011 Feb 17;6(2):e14711. doi: 10.1371/journal.pone.0014711 (PMC3040744; doi:10.1371/journal.pone.0014711)
Supplement: References S1 — (0.04 MB DOC) [file pone.0014711.s001.doc]

**Tumurbaatar SI**

Reference S1. Mailand N, Lukas C, Kaiser BK, Jackson PK, Bartek J, et al. (2002) Deregulated human Cdc14A phosphatase disrupts centrosome separation and chromosome segregation. Nat Cell Biol 4:317-322.

Reference S2. van de Wetering M, Oving I, Muncan V, Fong MJP, Brantjes H, et al. (2003) Specific inhibition of gene expression using a stably integrated, inducible small-interfering-RNA vector. EMBO Rep 6:609-615.
