# Supplementary material for: Human Cdc14B Promotes Progression through Mitosis by Dephosphorylating Cdc25 and Regulating Cdk1/Cyclin B Activity
Source: PLoS One. 2011 Feb 17;6(2):e14711. doi: 10.1371/journal.pone.0014711 (PMC3040744; doi:10.1371/journal.pone.0014711)
Supplement: Table S1 — Sequences of the siRNAs used in this study. All sequences are written from 5 to 3 orientation. Chemically synthesized siRNA duplexes were obtained from Eurogentec (siCdc14B), MWG (siGFP), and Dharmacon (siCdc14A and siCtrl corresponding to the non-targeting siRNA pool 1). Sequences of the non-targeting siRNA pool 1 are available from Dharmacon. (0.03 MB DOC) [file pone.0014711.s009.doc]

**Table S1. Sequences of siRNAs used in this study.**

| siRNA | sense sequence | antisense sequence |
| --- | --- | --- |
| siCdc14B-1 | GGGUGCCAUUGCAGUACAUTT | AUGUACUGCAAUGGCACCCTT |
| siCdc14B-2 | GAAGCAGCAUAUAGAAUAUTT | AUAUUCUAUAUGCUGCUUCTT |
| siCdc14B-3 | GAGCAGCCUUCUCCAAACUTT | AGUUUGGAGAAGGCUGCUCTT |
| siCdc14A | see Mailand et al. [Reference S1] | see Mailand et al. [Reference S1] |
| siGFP | GGCUACGUCCAGGAGCGCACCTT | GGUGCGCUCCUGGACGUAGCCTT |
| siCtrl | non-targeting siRNA pool 1 | non-targeting siRNA pool 1 |

All sequences are written from 5’ to 3’. Chemically synthesized siRNA duplexes were obtained from Eurogentec (siCdc14B), MWG (siGFP), and Dharmacon (siCdc14A and siCtrl corresponding to the non-targeting siRNA pool 1). Sequences of the non-targeting siRNA pool are available from Dharmacon.
