# Supplementary material for: Human Cdc14B Promotes Progression through Mitosis by Dephosphorylating Cdc25 and Regulating Cdk1/Cyclin B Activity
Source: PLoS One. 2011 Feb 17;6(2):e14711. doi: 10.1371/journal.pone.0014711 (PMC3040744; doi:10.1371/journal.pone.0014711)
Supplement: Table S3 — Sequences of primers used for RT-qPCR in this study. (0.03 MB DOC) [file pone.0014711.s011.doc]

**Table S3. Sequences of primers used for RT-qPCR in this study.**

|  | Forward primer sequence | Reverse primer sequence |
| --- | --- | --- |
| hCdc14A | GATGCTTCCTTTGGAAATTGC | TTTGCTTTTAGGATGTGGTCC |
| hCdc14B | AGGATGTATGATGCCAAACGC | GCTGCTGTCATCCTGTAATGC |
| actin | CGTCACCAACTGGGACGACA | CTTCTCGCGGTTGGCCTTGG |

The sequences of the DNA oligonucleotides are shown in 5’ to 3’ orientation.
